# DnaJ Proteins Regulate *WUS* Expression in Shoot Apical Meristem of Arabidopsis

**DOI:** 10.3390/plants10010136

**Published:** 2021-01-12

**Authors:** Tianqi Jia, Fan Li, Shuang Liu, Jin Dou, Tao Huang

**Affiliations:** School of Life Sciences, Xiamen University, Xiamen 361102, China; 21620150150523@stu.xmu.edu.cn (T.J.); 21620181153678@stu.xmu.edu.cn (F.L.); 21620201153217@stu.xmu.edu.cn (S.L.); doudoukuaile@yeah.net (J.D.)

**Keywords:** stem cell, WUS, DnaJ proteins

## Abstract

WUSCHEL (WUS) protein regulates stem cell function in shoot apical meristem of *Arabidopsis*. The expression of *WUS* gene is strictly regulated by developmental cues and environmental factors. As DnaJ domain-containing proteins, SDJ1 and SDJ3 have been proven to play an important role in transcriptional activation of promoter methylated genes. Here, we showed that three DnaJ domain-containing proteins including SDJ1 and SDJ3 can bind WUS protein as a complex, which further maintain the expression of *WUS* gene by binding to *WUS* promoter. We propose a model how DnaJ domain-containing proteins are involved in the self-regulation of *WUS* gene in stem cells maintenance of Arabidopsis.

## 1. Introduction

The continual propagation and differentiation of stem cells provide a basis for the postembryonic growth and development of plants. The stem cells in shoot apical meristem (SAM) of Arabidopsis are defined by the homeodomain proteins WUSCHEL (WUS), which is expressed in the organization center (OC). WUS protein can move to stem cell region where it can activate downstream gene *CLVATA3*, which conversely inhibit WUS expression, thereby maintaining stem cell homeostasis in SAM [1,2,3].

Previous studies have demonstrated that abiotic stresses can induce the accumulation of heat shock proteins (HSPs) at different molecular weights [4,5]. As a subfamily of heat shock protein, HSP40s are characteristic of the conserved DnaJ-domain that is responsible for interaction with HSP70s [6,7,8]. HSP40s can be divided into seven subfamilies according to the containing J-domains in Arabidopsis. DnaJA, DnaJB, and DnaJC subfamilies contain a typical J-domain that interacts with HSP70 proteins [9]. DnaJD, DnaJE, DnaJF, and DnaJG proteins are HSP70-independ because of the lacking of a typical J-domain [8,10,11]. HSP40/DnaJ-proteins are involved in the protein homeostasis by regulating protein folding/refolding, assembly, translocation, and stabilization under abiotic stress [10,12,13]. Previous researches have demonstrated the crucial roles of DnaJ-proteins in growth and development, hormone regulation, and acclimation of plants to abiotic stress [14,15,16,17,18,19]. As two members of DnaJD subfamily, SDJ1 and SDJ3 have been shown to interact with SUVH1 or SUVH3 protein as a SUVH-SDJ complex, which further mediates the transcriptional activation of promoter methylated genes [20,21]. SDJ1 protein also has been shown to be involved in flowering time control of Arabidopsis [18]. In this work, we showed that SDJ1 and SDJ3 proteins also interact with WUS protein as a complex which further regulate *WUS* expression by binding to *WUS* promoter.

## 2. Results and Discussion

### 2.1. WUS Interacts with DnaJ Proteins In Vivo and In Vitro

**Figure 1:**
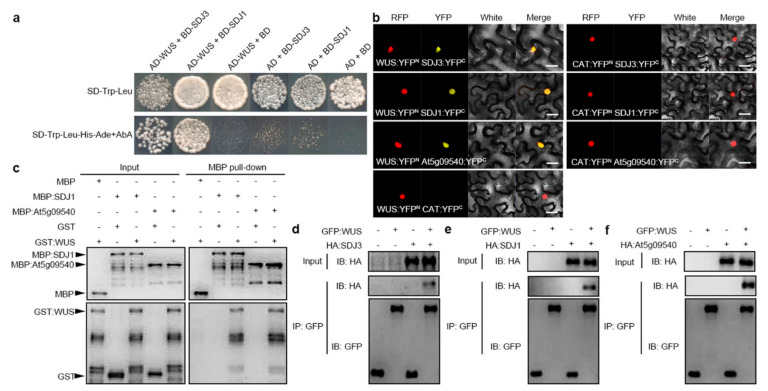
WUS protein interacts with DnaJ proteins (**a**) WUS protein interacts with SDJ1 and SDJ3 proteins in Yeast-two-hybrid assays. (**b**) Bimolecular fluorescence complementation in tobacco epidermal cells. WUS:YFPn protein in combination with SDJ3:YFPc, SDJ1:YFPc, and At5g09540:YFPc, respectively, was colocalized with Coilin:RFP protein in nucleus. CAT:YFPn and CAT:YFPc were used in control assays. Scale bar = 25 μm. (**c**) Input of recombinant proteins detected by immunoblotting with anti-GST antibody and anti-MBP antibody (Left) and pull down of GST-tagged WUS protein through MBP-tagged SDJ1 and MBP-tagged At5g09540 proteins detected by immunoblotting with anti-GST antibody (right). (**d**–**f**) Co-immunoprecipitation of GFP:WUS and HA:SDJ3 (**d**), GFP:WUS and HA:SDJ1 (**e**), GFP:WUS and HA:At5g09540 (**f**) detected by immunoblotting with anti-HA antibody.

**Figure 2:**
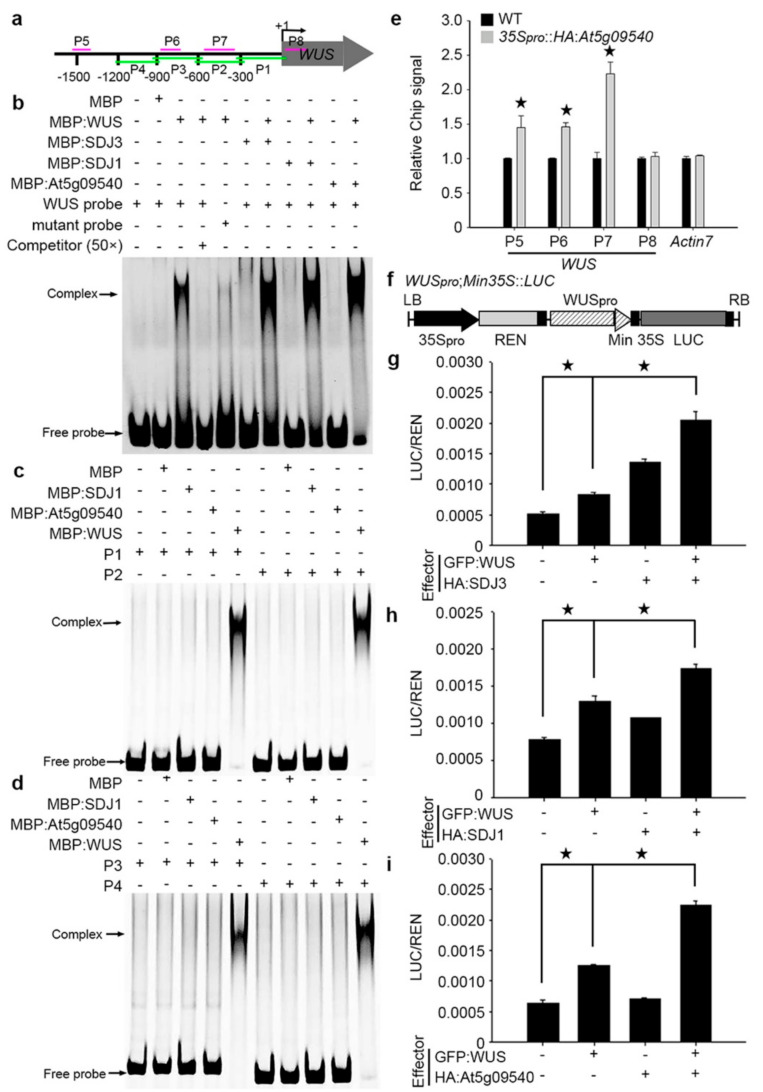
WUS-DnaJ complexes bind to *WUS* promoter and regulate *WUS* promoter activity. (**a**) Diagram of amplicon locations in *WUS* promoter. (**b**) DnaJ proteins bind to Texas red-labeled *WUS* promoter probe via the interaction with WUS protein as a complex in EMSA assay. The unlabeled probe was used as competitor. The TAAT box was substituted by CCCC in *WUS* mutant probe to abolish the binding of WUS protein. (**c**,**d**) SDJ1 and At5g09540 proteins failed to bind to P1(−312 bp–+25 bp) and P2 (−608 bp–−285 bp)regions (**c**) and P3 (−926 bp–−580 bp)and P4(−1203 bp–−895 bp) regions (**d**) of *WUS* promoter in EMSA assay. (**e**) Chromatin immunoprecipitation of At5g09540 protein with different regions (P5, P6, P7, P8) of *WUS* promoter and an *Actin7* promoter region in *35S_pro_::HA:At5g09540* plants. P5(−1570 bp–−1481bp), P6(−869 bp–−774 bp), and P7(−515 bp–−340 bp) regions contain TAAT box, but not P8 (+12 bp–+121 bp) region. The ChIP signal was calculated with three independent biological replicates, Student-t test, * *p* < 0.05. (**f**–**i**) LUC/REN activity in tobacco cells co-transformed with *WUSpro;min35S::LUC* reporter construct and indicated effectors. (**f**) Diagram of*WUSpro; Min35S::LUC* reporter construct. 35S: CaMV 35S promoter; WUSpro: 2.2 Kb *WUS* promoter (−1–−2200 bp); Min35S: 47 base pair 35S minimal element; REN: Renilla luciferase, LUC: firefly luciferase. LB/RB: T-DNA left or right border. The *WUS* promoter activity was significantly increased by WUS protein in combination with SDJ3 (**g**), SDJ1 (**h**), and At5g09540 (**i**), respectively. Error bar = mean ± SEM (*n* = 4 biological replicates). Student-t test, * *p* < 0.05.

When we used WUS protein as a bait to screen binding proteins from an *Arabidopsis* library by yeast-two-hybrid, one DnaJ-domain protein encoded by *At1g62970* was identified as a possible WUS-binding protein, which was already named as SDJ3. There are another two DnaJ-domain containing proteins encoded by *At5g64360* and *At5g09540* sharing high homologue with SDJ3 in *Arabidopsis* genome. The protein encoded by *At5g64360* was already named as SDJ1. SDJ1 and SDJ3 also interacted with WUS protein in yeast (Figure 1a). The interactions between SDJ1/SDJ3/At5g09540 and WUS were also confirmed by bimolecular fluorescence complementation (BiFC) assays in epidermal cells of tobacco (*Nicotiana benthamiana*). Strong YFP fluorescence was observed in nuclei when WUS-YFPn was co-transformed with SDJ1-YFPc, SDJ3-YFPc and At5g09540-YFPc, respectively (Figure 1b). The interactions between SDJ1/At5g09540and WUS were further confirmed by in vitro pull-down assay. The glutathione S-transferase (GST)-WUS, but not GST, bound the maltose-binding protein (MBP)-fused SDJ1, and MBP-At5g09540 proteins, respectively (Figure 1c). Therefore, DnaJ proteins can bind WUS protein as a complex independent of HSP70 proteins. Interactions *in planta* between SDJ1/SDJ3/At5g09540 proteins and WUS were also examined using co-immunoprecipitation assays. When HA-tagged SDJ1/SDJ3/At5g09540 proteins and GFP:WUS protein were transiently expressed in the epidermal cells of tobacco leaves, GFP:WUS protein bound SDJ1, SDJ3 and At5g09540, respectively (Figure 2d–f). Taken together, the interactions between SDJ1/SDJ3/At5g09540 proteins and WUS protein were confirmed with multiple methods.

### 2.2. DnaJ Proteins Function as a Co-Factor of WUS in Activating WUS Expression

Previous studies demonstrated that WUS protein can bind to TAAT motif in the promoter of downstream target genes [22]. *WUS* promoter also contains many TAAT motifs, indicating that WUS might bind to its own promoter for self-regulation. We examined whether SDJ1/SDJ3/At5g09540 proteins might bind to *WUS* promoter via interaction with WUS protein as a complex using electrophoretic mobility shift assay (EMSA). Our results showed that WUS protein can specifically bind to TAAT motif in a *WUS* promoter probe. The substitution of TAAT motif with CCCC abolished the binding to WUS protein to this promoter probe. WUS protein bound to its promoter more efficiently in the presence of SDJ1, SDJ3, and At5g09540 proteins, respectively (Figure 2a,b). SDJ1 and At5g09540 proteins on their own failed to bind to *WUS* promoter when different promoter regions were used in EMSA (Figure 2c,d). However, the binding of At5g09540 to different regions (P5–P7) of *WUS* promoter, but not coding region (P8), in *35S_pro_::HA:At5g09540* plants was confirmed using Chromatin immunoprecipitation (CHIP) assay (Figure 2e), demonstrating that At5g09540 protein can bind to *WUS* promoter via the interaction with endogenous WUS protein as a complex. 

To confirm the direct effects of WUS-DnaJ complex on the activities of *WUS* promoter, dual luciferase assays were conducted in tobacco leaf epidermal cells in which *WUS_pro_::LUC* construct was transformed in combination with the expression of GFP:WUS and HA-tagged SDJ1, SDJ3, and At5g09540 proteins, respectively. The luciferase activity was dramatically increased when GFP:WUS and SDJ1/SDJ3/At5g09540 proteins were combined in comparison with GFP, GFP:WUS or SDJ1/SDJ3/At5g09540 proteins alone (Figure 2f–i). These results demonstrated a positive role of WUS-SDJ1/SDJ3/At5g09540 complex in *WUS* promoter activity.

### 2.3. Disruption of DnaJ Proteins by Chimeric Repressor Silencing Technology Led to Premature Termination of SAM

**Figure 3:**
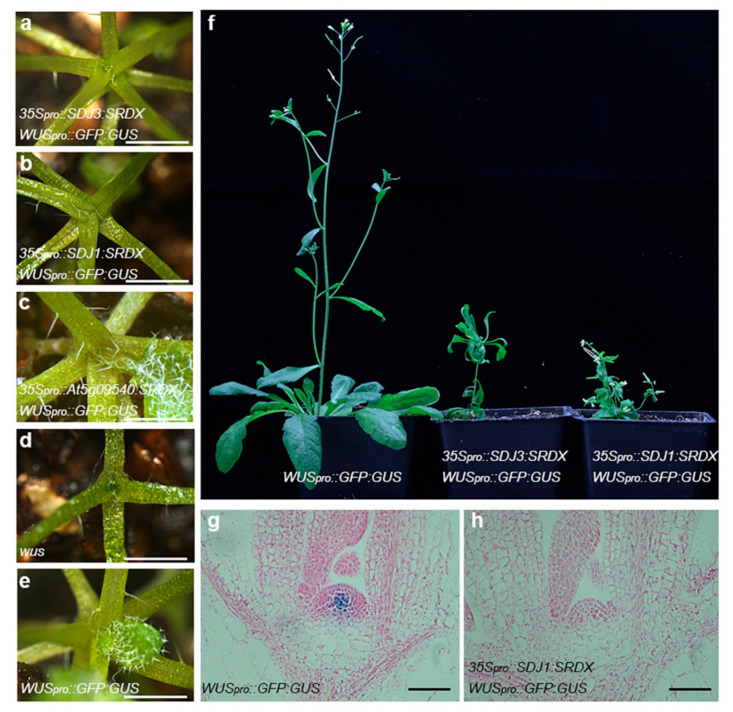
Overexpression of DnaJ-SRDX protein inhibits SAM development in Arabidopsis. (**a**–**e**) The premature termination of SAM in strong lines of *35S_pro_::SDJ3-SRDX WUS_pro_::GFP:GUS* plant (**a**), *35S_pro_::SDJ1-SRDX WUS_pro_::GFP:GUS* plant (**b**), *35S_pro_::At5g09540-SRDX WUS_pro_::GFP:GUS* plant (**c**), and *wus* mutant (**d**) in comparison with that of *WUS_pro_::GFP:GUS* control plant (**e**). (**f**) Phenotypes of weak lines of four-week-old *35S_pro_::SDJ3-SRDX WUS_pro_::GFP:GUS* plant and *35S_pro_::SDJ1-SRDX WUS_pro_::GFP:GUS* plant in comparison with *WUS_pro_::GFP:GUS* control plant. (**g**,**h**) *WUS* promoter activity in *35S_pro_::SDJ1-SRDX WUS_pro_::GFP:GUS* plant (**h**) in comparison with *WUS_pro_::GFP:GUS* plant (**g**) examined by GUS staining. Scale bar = 5 mm in (**a**–**e**), 50 μm in (**g**,**h**).

A transcriptional activator can be converted into a strong repressor by fusion with the ERF-associated amphiphilic repression (EAR) motif repression domain (SRDX), which then specifically suppress the expression of target genes by binding to their promoters [23,24,25]. This designated chimeric repressor gene silencing technology (CRES-T) can also be used to examine the protein–protein interactions and protein functions in growth and development of plants [26]. The *35S_pro_::SDJ1-SRDX*, *35S_pro_::SDJ3-SRDX and 35S_pro_::At5g09540-SRDX* transgenic plants were also created to examine the role of SDJ1/SDJ3/At5g09540 proteins in *WUS* expression and stem cell maintenance *in planta*. The strong *35S_pro_::SDJ3-SRDX* lines produced an umbrella structures on the top end of shoot, which is similar to the typical phenotype of *wus* mutant. The *35S_pro_::SDJ1-SRDX* plants and *35S_pro_::At5g09540-SRDX* plants also showed the premature termination of SAM (Figure 3a–f). GUS staining showed that the *WUS* promoter activity is significantly repressed in the SAM of *35S_pro_::SDJ1-SRDX WUS_pro_::GFP:GUS* plants (Figure 3g,h). Taken together, SDJ1/SDJ3/At5g09540 proteins can interact with WUS as a complex which further regulate the expression of *WUS* gene by binding to *WUS* promoter.

### 2.4. SDJ1 and SDJ3 Genes are Expressed in the SAM, RAM and Vascular Tissues of Leaf and Hypocotyl

**Figure 4:**
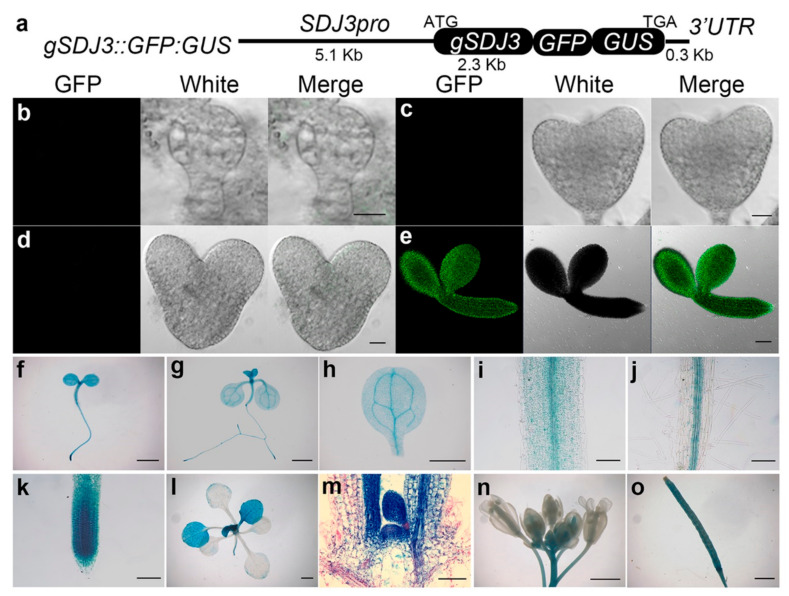
Expression of *SDJ3* gene in different organs at different developmental stages. (**a**) Diagram of *gSDJ3::GFP:GUS* construct. (**b**–**e**) SDJ3:GFP:GUS fusion protein is expressed at mature stage (**e**), but not global stage (**b**), heart stage (**c**), and torpedo stage (**d**) during the embryogenesis of *gSDJ3::GFP:GUS* plant by GFP fluorescence scanning. (**f**–**o**) Expression of SDJ3:GF:GUS fusion protein in 3-day-old seedling (**f**), 7-day-old seedling (**g**), cotyledon (**h**), hypocotyl (**i**), root (**j**), and RAM (**k**) of 7-day-old seedling, 14-day-old seedling (**l**), SAM of 14-day-old seedling (**m**), Flower (**n**), and Silique (**o**) examined by GUS staining. Scale bar= 10 μm in (**b**–**d**); 50 μm in (**e**,**m**); 1000 μm in (**f**–**l**,**n**) and o; 100 μm in (**i**–**k**).

**Figure 5:**
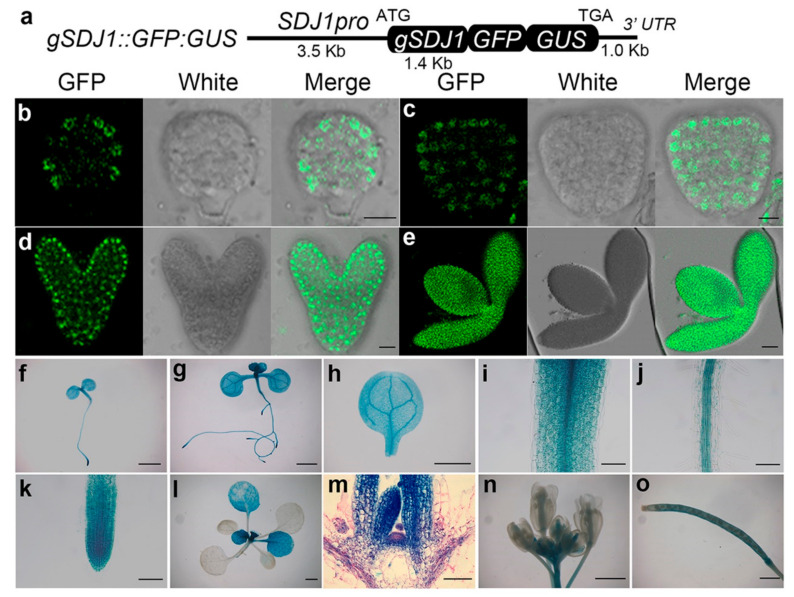
Expression of *SDJ1* gene in different organs at different developmental stages. (**a**) Diagram of *gSDJ1::GFP:GUS* construct. (**b**–**e**) SDJ1:GFP:GUS fusion protein is expressed at global stage (**b**), heart stage (**c**), and torpedo stage (**d**), and mature stage (**e**) during the embryogenesis of *gSDJ1::GFP:GUS* plant by GFP fluorescence scanning. (**f**–**o**) Expression of SDJ1:GFP:GUS fusion protein in 3-day-old seedling (**f**), 7-day-old seedling (**g**), cotyledon (**h**), hypocotyl (**i**), root (**j**), and RAM (**k**) of 7-day-old seedling, 14-day-old seedling (**l**), SAM of 14-day-old seedling (**m**), Flower (**n**), and Silique (**o**) examined by GUS staining. Scale bar= 10 μm in (**b**–**d**); 50 μm in (**e**,**m**); 1000 μm in (**f**–**l**,**n**) and o; 100 μm in (**i**–**k**).

**Figure 6:**
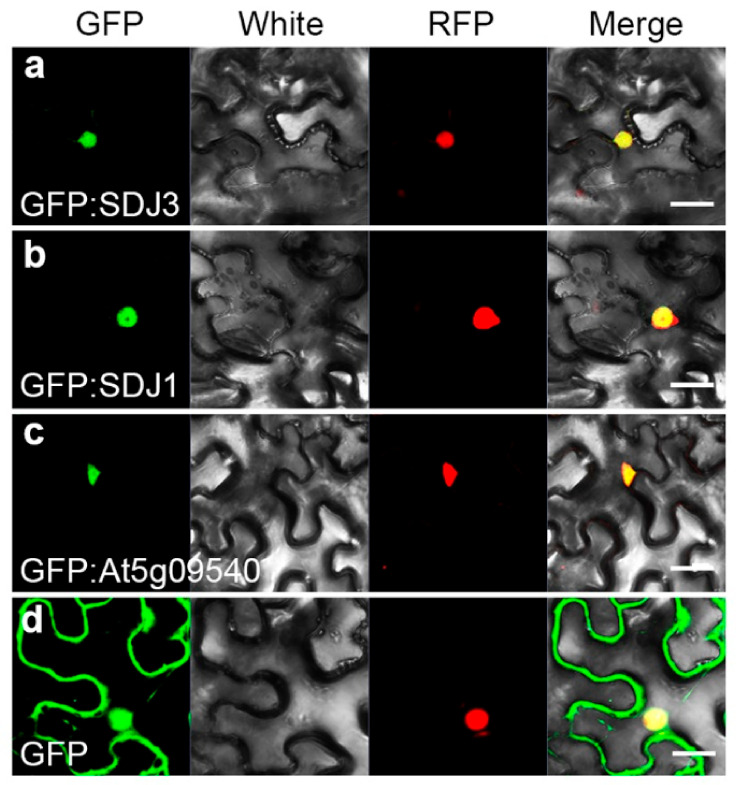
Localization of DnaJ proteins in nucleus. (**a**–**c**) The transiently expressed GFP:SDJ3, GFP:SDJ1, and GFP:At5g09540 proteins are colocalized with Coilin:RFP protein in nucleus in tobacco epidermal cells. (**d**) GFP protein is freely distributed in cytoplasm and nucleus. Scale bar = 25 μm.

To examine the expression pattern of *SDJ1* and *SDJ3* genes, *gSDJ3::GFP:GUS* and *gSDJ1::GFP:GUS* transgenic plants were created to express SDJ3:GFP:GUS and SDJ1:GFP:GUS fusion proteins, respectively, under the control of their own promoters (Figure 4a and Figure 5a). The expression of SDJ3:GFP:GUS protein was only detected at mature stage during embryogenesis (Figure 4b–e). However, SDJ1:GFP:GUS protein was already expressed from globular stage during embryogenesis (Figure 5b–e). After germination, GUS staining showed that SDJ3:GFP:GUS protein and SDJ1:GFP:GUS protein are mainly expressed in SAM, RAM and vascular tissues of leaf and hypocotyl, as well as younger but not fully differentiated leaves (Figure 4f–o and Figure 5f–o). In addition, SDJ1/SDJ3/At5g09540 in fusion with GFP protein was transiently expressed and located in nucleus of tobacco epidermal cells (Figure 6).

### 2.5. DnaJ Proteins Contribute to the Expression of WUS mRNA 

**Figure 7:**
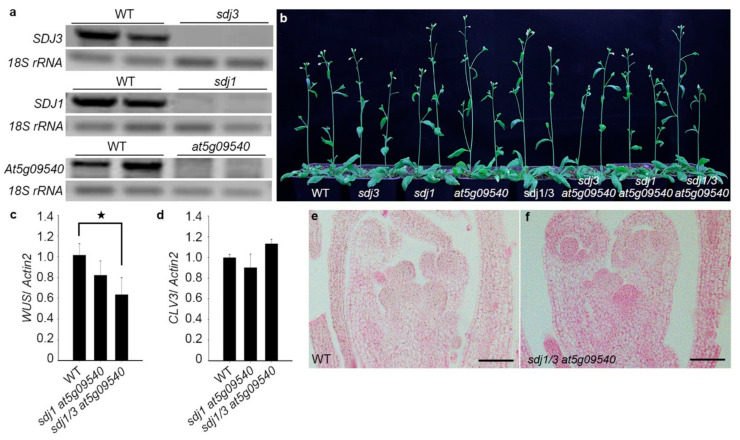
DnaJ protein regulate *WUS* mRNA expression. (**a**) RT-PCR analysis of of relative genes in *sdj3*, *sdj1*and *at5g09540* mutants. (**b**) Growth phenotype of WT, *sdj3*, *sdj1*, *at5g09540*, *sdj3 sdj1*, *sdj1 at5g09540*, *sdj3 at5g09540*, *and sdj3 sdj1 at5g09540* plants. (**c**,**d**) *WUS* mRNA level and *CLV3* mRNA level in WT and *sdj3 sdj1 at5g09540* plants by quantitative RT-PCR analysis. Student-t test, * *p* < 0.05 (**e**,**f**) SAM histology of WT and *sdj3 sdj1 at5g09540* plants.

The binding of WUS protein to its own promoter might be necessary for keeping the normal expression level of *WUS* gene. To explore the roles of *SDJ1/SDJ3/At5g09540* genes in growth and development of Arabidopsis, the *sdj1*, *sdj3*, and *at5g09540* knockout mutants were identified by RT-PCR analysis of the relative endogenous *SDJ1/SDJ3/at5g09540* expression (Figure 7a). The *sdj1 sdj3 at5g09540 triple* mutant was also created by genetic crossing. The *sdj1 sdj3 at5g09540* mutant grew normally as WT plants under normal growth condition possibly because other redundant DNAJ domain proteins might also interact with WUS protein as a complex, which also might be involved in *WUS* expression (Figure 7b). Actually, the DnaJ-domain protein encoded by *At4g11930* also interacted with WUS protein in BiFC analysis (Figure S1), indicating that more proteins from DnaJ-domain protein family might interact with WUS protein. *WUS* mRNA in three-day-old *sdj1 sdj3 at5g09540* mutant was decreased to a lower level in comparison with that of *WT* plants, suggesting that SDJ1/SDJ3/At5g09540 proteins contribute to the expression of *WUS* mRNA (Figure 7c). In addition, as a downstream target of WUS protein, the expression of *CLV3* was not significantly changed in *sdj1 sdj3 at5g09540* mutant (Figure 7d). The inflorescence meristem morphology of *sdj1 sdj3 at5g09540* mutant was not significantly altered in comparison with WT (Figure 7e,f), indicating that SAM development can tolerate a variation range of *WUS* expression. Taken together, DnaJ proteins can maintain *WUS* expression by interacting with WUS protein as a complex which further bind to *WUS* promoter in SAM of Arabidopsis. We also cloned ortholgues of *WUS* and *SDJ1* from *Brassica napus*. The interaction between BnWUS (CDY30337) and BnSDJ1 (XP_013722826) was also confirmed in BIFC analysis (Figure S2). Therefore, DnaJ proteins might be universally employed as WUS-binding protein to regulate stem cell function in SAM in plant kingdom.

## 3. Materials and Methods

### 3.1. Plant Materials and Transformation

All constructs were introduced into Col-0 by *Agrobacterium tumefaciens* PMP90 strain according to the reported floral-dip protocol [27]. At least three independent transgenic lines were used for subsequent analyses. The *sdj3* mutant (Salk_016979), *sdj1* mutant (Salk_021078), a*t5g09540* mutant (SAIL_735_A07), and *wus* mutant (Salk_114398) were ordered from the European Arabidopsis Stock Centre. All plants were grown in soil at 22 °C under long days (16 h light/8 h dark) with white fluorescent light (120 μmol·m^−2^·s^−1^).

### 3.2. Plasmids Construction for Transgenic Plants

The CaMV *35S* promoter in the *pB7GW2* vector was replaced with the putative 5.1 Kb *SDJ3* promoter (−1 bp–−5100 bp) to create *pB7GW2_SDJ3_pro_*. The *SDJ3* genomic fragment (from translation start codon to stop codon) and 0.3 Kb downstream region after the coding region were cloned into the *pDONR201_GFP:GUS* vector to create *pDONR201*_*gSDJ3:GFP:GUS* for the in-frame fusion of the *GFP:GUS* with the *SDJ3* gene. The *gSDJ3:GFP:GUS* usion gene was further cloned into *pB7GW2_SDJ3_pro_* to create the *gSDJ3:GFP:GUS* construct by LR clonase (Invitrogen, Carlsbad, CA, USA). *gSDJ1:GFP:GUS* construct containing 3.5 Kb promoter region, 1.392 Kb genomic region and 1.0 Kb downstream region was also created by the same strategy. The *SDJ1*, *SDJ3* and *At5g09540* cDNAs were amplified with *Xba*I (5′-end) and *Spe*I (3′-end) sites and cloned into *pDONR201* to create *pDONR201-SDJ1/SDJ3/At5g09540*, respectively. The *GFP* cDNA was inserted at the unique *Xba*I site and unique *Spe*I site in *pDONR201-SDJ1/SDJ3/At5g09540* to create the *pDONR201-GFP:*SDJ1/SDJ3/*At5g09540*, respectively. The HA tag and SRDX domain (LDLDLELRLGFA) were also cloned into the *pDONR201-SDJ1/SDJ3/At5g09540* to create *pDONR201_HA:SDJ1/SDJ3/At5g09540* and *pDONR201_ SDJ1/SDJ3/At5g09540:SRDX*, respectively. These genes harbored in the *pDONR201* was further cloned into *pK2GW7* to create *35S_pro_::GFP:SDJ1/SDJ3/At5g09540*, *35S_pro_::HA:SDJ1/SDJ3/At5g09540* and *35S_pro_:: SDJ1/SDJ3/At5g09540:SRDX* constructs, respectively.

### 3.3. Protein Purification

The *WUS* cDNA was cloned into pGEX vector and *pMBP-c* vector, respectively. *SDJ1/SDJ3/At5g09540* cDNAs were also cloned into *pMBP-c* vector, respectively. The target proteins were expressed in *E. coli* BL21 with the induction of 0.25–1.0 mM isopropyl β-d-1-thiogalactopyranoside (IPTG) for 9 h at 16 °C or 22 °C and purified using glutathione resin (Thermo Fisher Scientific, Waltham, MA, USA) or amylose resin (New England Bio-Labs, Ipswich, MA, USA).

### 3.4. Yeast Two-Hybrid Assay

*WUS* cDNA was cloned into *pGADT7* vector. *SDJ1* and *SDJ3* cDNAs were cloned into *pGBKT7* vector. The bait and prey vectors were transformed into Y2HGold yeast strain following the manufacturer’s instructions (Clontech, Palo Alto, CA, USA).

### 3.5. In Vitro Pull-Down Assay

MBP:SDJ1, MBP:At5g09540 and MBP were immobilized on amylose resin and incubated with GST:WUS or GST, respectively, in the reaction buffer (5% glycerol, 1% triton-100, 50 mM Tris-HCl(pH 7.4), 150 mM NaCl, 10 mM NaF, 1 mM Na_3_VO_4_, 10 mM leupeptin, 1 mM phenylmethylsulfonyl fluoride (PMSF)) for 2 h at 4 °C with rotation. The amylose resin was then washed three times and subjected to immunoblot analysis to detect the precipitated GST:WUS protein using an anti-GST antibody (Cell signaling Technology, Shanghai, China). All pull-down assays were repeated three times with the similar results.

### 3.6. Bimolecular Fluorescence Complementation (BiFC) Assays

Full-length Arabidopsis *WUS and Brassica napus WUS* were cloned into *p2YN* vector containing N-terminal half of YFP to generate the fusion proteins of WUS-YFPn and BnWUS-YFPn, respectively. *SDJ1*, *SDJ3*, *At5g09540*, *At4g11930* and *BnSDJ1* cDNAs were cloned into *p2YC* vector containing C-terminal half of YFP to produce the fusion proteins of SDJ1-YFPc, SDJ3-YFPc, At5g09540-YFPc, At4g11930-YFPc, and BnSDJ1-YFPc, respectively. These constructs together with *35S_pro_::Coilin:RFP* were introduced into *Nicotiana benthamiana* leaves through *Agrobacterium* infiltration so that Coilin-RFP protein can be localized at nucleus. Each BiFC experiment was repeated with four independent biological replicates. YFP fluorescence and RFP fluorescence were recorded using a confocal laser scanning microscope (Zeiss LSM 780) with excitation/emission values of 513 nm/495–545 nm and 561 nm/578–636 nm, respectively.

### 3.7. Co-Immunoprecipitation

*35S_pro_::GFP* and *35S_pro_::GFP:WUS* in combination with *35S_pro_::HA:SDJ3*, *35S_pro_::HA:SDJ1*, or *35S_pro_::HA:At5g09540* were introduced into *Nicotiana benthamiana* leaves through *Agrobacterium* infiltration to examine the interactions of GFP:WUS with HA:SDJ3, HA:SDJ1 and HA:At5g09540, respectively. The leaves were harvested two days after infiltration and frozen in liquid nitrogen for the isolation of nuclei. The isolated nuclei were lysed with reaction buffer (50 mM Tris-HCl, pH 8.0, 10 mM EDTA, 1%Triton X-100, 0.1 mM PMSF, 1×Protease inhibitors cocktail) by incubation on ice for 30 min. Clear lysates were mixed with diluting buffer (50 mM Tris-HCl, pH 7.5, 150 mM NaCl, 3 mM DTT, 2 mM NaF and 1 mM NaVO3) and immunoprecipitated with GFP-Trap^®^ agarose beads (ChromoTek). The beads were washed three times with the diluting buffer. The proteins recovered from the beads were subjected to immunoblot analysis using anti-GFP antibody (Cell Signaling Technology) or anti-HA antibody (Cell Signaling Technology).

### 3.8. Chromatin Immunoprecipitation (ChIP)

The 2 g of 4-day-old WT and *35S_pro_::HA:At5g09540* seedlings were harvested and fixed with 30 mL 1% formaldehyde under vacuum following addition of 2 mL 2M glycine to stop the crosslinking. Nuclei were isolated from tissue powder with 30 mL isolation buffer (10 mM Tris-HCl (pH 8.0), 0.4 M sucrose, 10 mM MgCl_2_, 5 mM 2-mercaptoethano (β-ME), 1% Triton-X100, 0.1 mM PMSF, 1×Protease inhibitors cocktail) and lysed with 300 μL lysis buffer (50 mM Tris-HCl (pH 8.0), 10 mM EDTA, 1% SDS, 0.1 mM PMSF, 1×Protease inhibitors cocktail). Chromatin was sheared to an average size of 200 bp–500 bp by sonication for total 550 s on a Covaris M220 Focused-ultrasonicator. About 10 % of sonicated chromatin was collected as Input. The residue of sonicated chromatin was diluted with 10 times volume of dilution buffer (16.7 mM Tris-HCl (pH 8.0), 1.2 mM EDTA, 167 mM NaCl, 1% Triton-X100, 0.1 mM PMSF, 1×Protease inhibitors cocktail) and precleared by incubation with 30 µL of Dynabeads^®^ protein A (Invitrogen). Then precleared chromatin was incubated with 30 µL of Dynabeads^®^ protein A coupled to an anti-HA antibody or anti-GFP antidody overnight at 4 °C with gentle agitation. The precipitated DNA was used as template for real-time RT-PCR analysis. The relative quantity (RQ) for each immunoprecipitated promoter region was calculated as ChIP/Input ratio. The relative quantity using Anti-HA antibody (RQ_HA_) was normalized to the relative quantity using Anti-GFP antibody (RQ_GFP_) as the relative enrichment of a promoter region. Four different loci in *WUS* promoter region were analyzed. *Actin7* was also used as a negative control. The primers used for ChIP-qPCR are listed in Supplemental Table S1. All ChIP experiments were performed with four independent biological replicates.

### 3.9. Electrophoretic Mobility Shift Assay (EMSA)

EMSA reactions were performed in a total volume of 10 μL of binding buffer (10 mM HEPES (PH 7.8), 20 mM KCl, 1 mM MgCl_2_, 21 mM DTT, 7.5% Glycerol, 0.5% Triton-100, 300 ng carrier DNA, 0.02% BSA) containing *WUS* promoter probes, MBP:WUS and MBP: SDJ1/SDJ3/At5g09540 at 22°C for 30 min. The *WUS* promoter probe (CTAGAATGAATAATAAAAAAAGTGAAAACCGTTTGATCATAA) containing a TAAT motif was labeled with Texas Red at 3′end. The unlabeled probe was used for competition experiments. The probe with the TAAT motif replaced by CCCC were used as negative control. The EMSA reaction products were resolved on 8% polyacrylamide gel and visualized using a ChemiDoc XRS molecular imaging system (Bio-Rad, Hercules, CA, USA).

### 3.10. Transactivation Assay in Tobacco

A 47 base pair minimal 35S promoter was amplified and cloned with *Spe*Ⅰ and *Xba*Ⅰ sites into *pGreenII-0800-LUC* to generate *pGreenII-0800-mini 35S-LUC* vector. The 2.0 Kb *WUS* promoter (−126 bp–−2127 bp) was cloned with *Kpn*Ⅰ and *Bam*HⅠ sites into *pGreenII-0800-mini 35S-LUC* vector to create *pGreenII-0800-WUS_pro_-mini 35S-LUC*. The dual-luciferase reporters in combination with different effectors (*35S_pro_::GFP*, *35S_pro_::GFP:WUS*, *35S_pro_::HA:SDJ3*, *35S_pro_::HA:SDJ1*, *35S_pro_::HA:At5g09540*) were introduced into *Nicotiana benthamiana* leaves through Agrobacterium infiltration. The activities of firefly luciferase (LUC) and Renilla luciferase (REN) were examined three days after infiltration using a Dual Luciferase Assay kit (Promega, Madison, WI, USA) on a luminometer (GloMax^®^20/20, Promega). The LUC activity was normalized to the REN activity (LUC/REN), and three replicate assays were performed with independent biological samples.

### 3.11. Subcellular Localization of DnaJ Proteins

*35S_pro_::GFP:SDJ3*, *35S_pro_::GFP:SDJ1*, and *35S_pro_::GFP:At5g09540* constructs in combination with *35S_pro_::Coilin:RFP* construct were introduced into *Nicotiana benthamiana* leaves through *Agrobacterium* infiltration. Then the colocalization of GFP:SDJ1, GFP:SDJ3 and GFP:At5g09540 proteins with Coilin:RFP protein in nucleus was examined using a confocal laser scanning microscope (Zeiss LSM 780) three days later after *Agrobacterium* infiltration.

### 3.12. RNA Isolation and Real-Time RT-PCR

Total RNA extraction, reverse transcription and qRT-PCR were performed as a previous report [28]. Three replicate assays were performed with independent RNA samples. All primers used for RT-PCR analysis are listed in Supplemental Table S1.

### 3.13. Histochemical Localization of GUS Activity

The histological analysis of β-glucuronidase activity was performed as the reported protocol [29]. GUS staining was performed with three independent transgenic lines with similar results.

### 3.14. Accession Numbers

Sequence data from this article can be found in the Arabidopsis Genome Initiative under the following accession numbers: *WUS* (At2g17950), *SDJ3* (At1g62970), *SDJ1* (At5g64360), At5g09540, At4g11930.

## Data Availability

Data is contained within the article or supplementary material.

## Supplementary Materials

The following are available online at https://www.mdpi.com/2223-7747/10/1/136/s1, Figure S1: At4g11930 interacts with WUS protein in tobacco epidermal cells in BiFC assay.,Figure S2: BnSDJ1 interacts with BnWUS protein in tobacco epidermal cells in BiFC assay, Table S1: The primers used in the experiment.
