# Supplementary material for: DnaJ Proteins Regulate WUS Expression in Shoot Apical Meristem of Arabidopsis
Source: Plants (Basel). 2021 Jan 12;10(1):136. doi: 10.3390/plants10010136 (PMC7827474; doi:10.3390/plants10010136)
Supplement: Supplementary file 1 [file plants-10-00136-s001.pdf]

Table S1. The primers used in the experiment.

Genes	Sequences	
SDJ3	5'-ATGATGAGCCATGGTGGTGG	for RT-PCR
	5'-TCAATCAACCTCAATTACTT	
SDJ1	5'-ATGGTAGGCGGACGCGGCGG	
	5'-CTAAGTAGCCTTGATCTTAT	
At5g09540	5'-ATGGTAAGCGACGGCGGCGG	
	5'-TTAATCATCATCAGAAATTG	
18S rRNA	5'-TCAACTTTCGATGGTAGGATAGTG	
	5'-CCGTGTCAGGATTGGGTAATTT	
WUS	5'-AAGCCATATCCCAGCTTCAA	for qRT-PCR
	5'-CCATCCTCCACCTACGTTGT	
Actin2	5'-GGTGGTTCCATTCTTGCTTC	
	5'-GAAACGCAGACGTAAGTAAAAAC	
WUSpro-P5	5'-ATCCTTAGATTGTTTTCCAA	for Chip-qPCR
	5'-ACACAACGTGCAAAGAGTAT	
WUSpro-P6	5'-ACGGTTTTCACTTTTTTTAT	
	5'-ATTCTAAAAGATCGTTATGA	
WUSpro-P7	5'-ACAAATTTTTTCATAAAAAT	
	5'-AAGGATATAAGAAAGAAAAA	
WUSpro-P8	5'-ACAGCATCAGCATCATCATC	
	5'-GTGTCCACCTCGTGCTGGTC	
Actin7	5'-TCTATGGAAACATCGTTCTC	
	5'-GAGGGATGCAAGGATTGATC	
pWUS-F Texas Red	5'-TTATGATCAAACGGTTTTCACTTTTTTTATTATTCATTCTAG	for EMSA
pWUS-F	5'-TTATGATCAAACGGTTTTCACTTTTTTTATTATTCATTCTAG	
pWUS-R	5'-CTAGAATGAATAATAAAAAAAGTGAAAACCGTTTGATCATAA	
pWUStu-F Texas Red	5'- TTATGATCAAACGGTTTTCACTTTTTTTGGGGTTCATTCTAG	
pWUStu-R	5'-CTAGAATGAACCCCAAAAAAAGTGAAAACCGTTTGATCATAA	
WUSpro-P1	5'-CCAAGCACATTTTTCAATAGGGTTTTG	
	5'-GATGCTGATGCTGTGGCG	
WUSpro-P2	5'-CCCTCCTTTGTCCCTTTCCATTTTC	
	5'-CAAAACCCTATTGAAAAATGTGCTTGG	
WUSpro-P3	5'-CACCTATGTGTCATGACTCATGACC	
	5'-CATGAAAATGGAAAGGGACAAAGG	
WUSpro-P4	5'-GTCATATTCACTAAAACAAAGCCCTTC	
	5'-CCTTATGGTCATGAGTCATGACAC	

Supplemental Figure 1

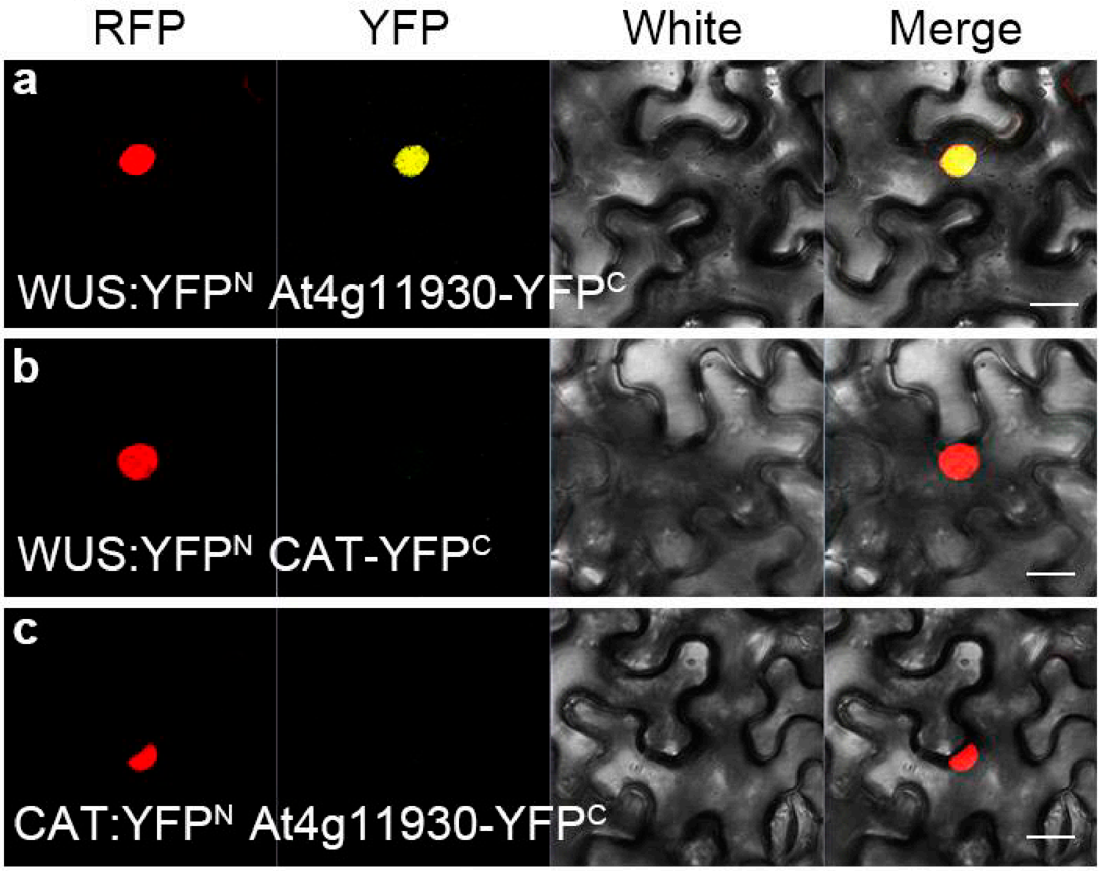

Figure S1. At4g11930 interacts with WUS protein in tobacco epidermal cells in BiFC assay. (a-c) Bimolecular fluorescence complementation in tobacco epidermal cells. WUS:YFPn protein in combination with At4g11930:YFPc was colocalized with Coilin:RFP protein in nucleus. Interactions between WUS-CAT and CAT-At4g11930 were not detected. Scale bar= 25 μm.

Supplemental Figure 2

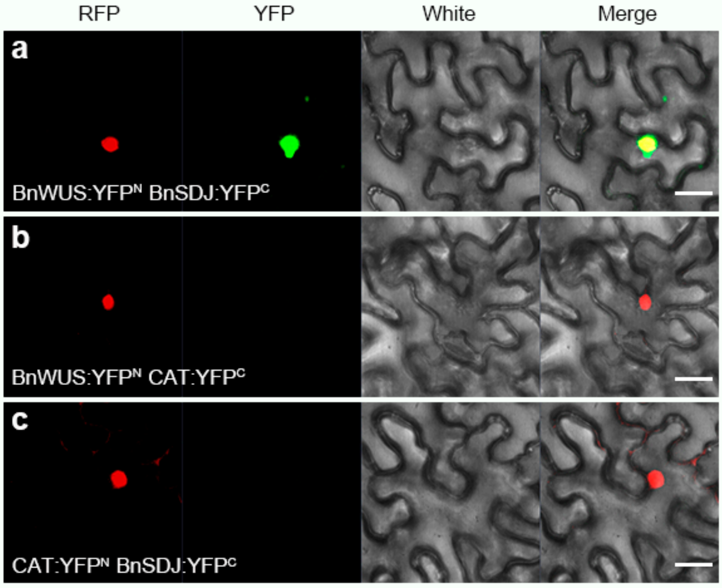

d

```
SDJ1            MVGG-----RGGVGGGGGNRVEADQWLVTSEKLLASSDLQGAKTFAIRACEADPTRAEAA      55
BnSDJ1          MVGGGDDGRGGAAGSRGGNKAEADRLLVTSEKLLSSSDFQGAKASAIRACKSDPTLAEAA      60
                ****      *..*. ***:.***: ********:***:****: *****::*** ****

SDJ1            DYILAICDILLAGETRLGDSNLPDWYAVLRLGRLAQNPEHVATQYRRLALLLNPSVNRLP      115
BnSDJ1          DSILTICDVLIASEIRLGNSKLPDWYAALRLGRLAQNPEHVATQYRRLALLLNPSVNRFP      120
                * **:***:*:*.* ***:*:******.******************************:*

SDJ1            FADRALKIVSDAWFVLSDPFKKSFYDRELQLSQLGQSGFHPQTQSHQNFQWEPSSSTAVY      175
BnSDJ1          LADQAFKVVSDAWHVLSDPSRKSLYDQEVQLSQLVQ------PQQKQRFIWKPSQSTTKV      174
                :**:*:*:*****.***** :**:**:*:***** *      *.:*.* *:**.**:

SDJ1            -----------PPP-RSQSQAGTSADPMATSFWTACPYCFVLFEYPKAYEECTLKCQECR      223
BnSDJ1          VAVSQHPPSVAPPATSTVGYPQRRSDPTATSFWTACPYCFVLFEYPKAYEECVLRCQDCR      234
                           **   : .     :** ************************.*:**:**

SDJ1            RAFQAVTIPKPPVDKKDDEDVYFCSWALFPLGFSGEFQAPSWSPISPLFACPLQKLDGE-      282
BnSDJ1          RAFQAVTIQKPPVEGK-GEDVYFCSWSVFPLGFSGEFKAPSWSPISPLIACPLQMVDDER      293
                ******** ****: * .*********::*********:**********:***** :*.*

SDJ1            -PKKRKEPGPPRIYYDDDDIYVAISDGDDAGDDGDWQDVVQVNKKTNVGKGKETYVRSNK      341
BnSDJ1          TTKKRKEPAPPRFFYDDDDVYVAISEDDHEENEPS-DNCQDELQAKNAGKGKEKLVRSNK      352
                  ******.***::*****:*****:.*.  :: . ::  :  : .*.*****. *****

SDJ1            KHGVE----EVQNVESISSASNHAVSIASNAAAVGSSTGVMSKPGSYITRKRMGTGAKNL      397
BnSDJ1          KRGADKAGKGIQHVESIGGASNHAASIASYVTPVGSAS--MS--KPMSSKKRTGTGAKNL      408
                *:*.:     :*:****..*****.**** .: ***::  **      ::** *******

SDJ1            GRLDLNVEFSNEVEEPGVAGGGNAGNGIGSNREVDNMEGIGFFEGLDEFLNSLPILSVVG      457
BnSDJ1          GRLDLNVEFSNEVEEPAVAGGRNEGNGLGSNREVDNMEGIGFFEDLDEFLSSLPIFSVDG      468
                ****************.**** * ***:****************.*****.****:** *

SDJ1            DDKIKAT     464
BnSDJ1          DDKIKAT     475
                *******
```

Figure S2. BnSDJ1 interacts with BnWUS protein in tobacco epidermal cells in BiFC assay. (a-c) Bimolecular fluorescence complementation in tobacco epidermal cells. BnWUS:YFPn protein in combination with BnSDJI:YFPc was colocalized with Coilin:RFP protein in nucleus. Interactions between BnWUS-CAT, or CAT-BnSDJ1 were not detected. Scale bar= 20 μm.

(d) Amino acid sequence alignment of BnSDJ1 (XP_013722826) from *Brassica napus* and SDJ1 from *Arabidopsis thaliana* using Clustal Omega.
